# Molecular detection of zoonotic RNA viruses in guinea pigs (*Cavia porcellus*) from small-scale family farming in the region of Cusco, Peru

**DOI:** 10.1016/j.onehlt.2026.101335

**Published:** 2026-01-20

**Authors:** Abel E. Quispe, Renzo Vera, Josimar Quiñones, José Angulo-Tisoc, César Lázaro, Alberto Manchego, Milagros Lostaunau, Edgar Valdez, Miguel Rojas, Dennis A. Navarro-Mamani

**Affiliations:** a Laboratorio de Inmunología, Facultad de Medicina Veterinaria, Universidad Nacional Mayor de San Marcos, Lima, Peru; b Laboratorio de Virología, Facultad de Medicina Veterinaria, Universidad Nacional Mayor de San Marcos, Lima, Peru; c Instituto Veterinario de Investigaciones Tropicales y de Altura, Sede Maranganí. Facultad de Medicina Veterinaria, Universidad Nacional Mayor de San Marcos, Cusco, Peru; d Laboratorio de Farmacología y Toxicología Veterinaria, Facultad de Medicina Veterinaria, Universidad Nacional Mayor de San Marcos, Lima, Peru; e Laboratorio de Sanidad Animal Atilio Pacheco Pacheco, Facultad de Agronomía y Zootecnia, Universidad Nacional de San Antonio Abad del Cusco, Cusco, Peru

**Keywords:** Guinea pig, *Cavia porcellus*, Zoonotic RNA viruses, Andean region, Peru

## Abstract

Emerging zoonotic diseases are frequently associated with close human-animal interactions in small-scale farming systems. Guinea pigs (*Cavia porcellus*) are widely raised for food in the Andean region, often under poor sanitary conditions; however, little is known about their role as reservoirs of enteric viruses with zoonotic potential. This study aimed to detect zoonotic RNA viruses in intestinal samples from guinea pigs raised on small-scale family farms in the Cusco region of Peru. A total of 34 intestinal tissue samples from adult guinea pigs showing gastrointestinal lesions were analyzed by reverse transcription polymerase chain reaction (RT-PCR) and nested PCR for the molecular detection of Coronavirus (CoV), Rotavirus A (RVA), *Mammalian orthoreovirus* (MRV), and Kobuvirus (KoV). Positive amplicons were sequenced and analyzed phylogenetically to confirm the PCR assays. Overall, 91.18% (31/34) of samples tested positive for at least one virus. RVA was the most frequently detected (58.82%), followed by CoV (29.41%), MRV (23.53%), and KoV (23.53%). Single-virus infections accounted for 20 cases and co-infections were identified in 11 cases. RVA was the most frequently detected, both in single (*n* = 9) and co-infections (*n* = 11). KoV detection was predominantly associated with co-infections rather than single infections. These findings provide the first molecular evidence of multiple zoonotic RNA viruses in guinea pigs from small-scale farming in Peru, highlighting their potential role as reservoirs in zoonotic transmission cycles. Enhanced surveillance and improved farm-level biosecurity are essential to mitigate risks of viral emergence in these traditional farming systems.

## Highlights

• First molecular evidence of zoonotic RNA viruses in guinea pig farming.
• High-frequency detection (91.18%) of zoonotic RNA viruses in guinea pig.
• Rotavirus A was the most frequently detected in single and co-infections.

## 1 Introduction

Emerging infectious diseases in humans are predominantly zoonotic, accounting for at least 75% of cases [1], [2]. These diseases occur more frequently in low- and middle-income countries, driven by factors such as close contact between humans and animals, limited disease surveillance, and under-resourced healthcare systems [3], [4]. In addition, human-induced environmental changes that increase contact with animals elevate the risk of virus transmission, highlighting the urgent need for global surveillance of viruses in animal farming systems [5]. Examples of zoonotic pathogens that have undergone spillover events resulting in efficient and sustained human-to-human transmission include Nipah virus, SARS coronavirus, hantaviruses, Ebola, Marburg virus, and Hendra virus [3], [6].

The NCBI/GenBank database contains about 299,000 farmed-mammal-associated viral sequences of which 83.4% correspond to RNA viruses, and 38.5% of these are known to be zoonotic [5]. RNA viruses pose particularly high zoonotic risks because they can emerge and spread rapidly, and a statistical analysis of 146 livestock viruses indicated that the ability of a virus to replicate in the cytoplasm (without nuclear entry) is the strongest single predictor of cross-species transmission and the ability to infect humans [7]. Recently in China, many viruses associated with human infection have been reported in guinea pig including members of the *Flaviviridae*, *Circoviridae*, *Orthomyxoviridae*, *Pneumoviridae*, *Spinareoviridae* and *Parvoviridae* [8]. Notable examples included *Mammalian orthoreovirus*, Japanese encephalitis virus, Influenza A virus and Rotavirus A [8]. Therefore, studying viruses in farm animals is critical for anticipating, understanding, and mitigating the risks associated with emerging zoonotic diseases.

In this context, the use of guinea pigs as companion animals and research models worldwide, and livestock in the Andean region makes them a potential reservoir for occupational zoonotic transmission [9]. Guinea pig farming is quite common and remains an essential food source and income generation for rural communities. Peru is a leading global producer, with over 25 million guinea pigs raised under traditional and small-scale family farming systems [10], [11]. Most guinea pig farms suffer from inadequate infrastructure and low technification, leading to poor animal health management and increased disease spread due to insufficient biosecurity and sanitation measures [11]. These deficits increase the risk of zoonotic pathogen transmission between animals and humans, especially in close-contact farming systems.

The intensive breeding of farmed animals creates conditions that can facilitate virus spillover from animals to humans, underscoring the importance of close monitoring. In addition, guinea pigs have been shown to carry a high diversity of viruses, including zoonotic pathogens, suggesting their potential role as intermediate hosts in transmission chains. Notably, domestication status has been identified as the strongest predictor of viral sharing between mammals and humans, with domesticated mammals harboring up to eight times more zoonotic viruses than wild species [1], [2]. Therefore, this study aimed to perform the molecular detection of zoonotic RNA viruses in intestinal samples from guinea pigs raised on small-scale family farms in the Cusco region of Peru.

## 2 Methodology

### 2.1 Study area

The current study was conducted in the Cusco region, the southeast Andean highlands of Peru. The predominant climate is semi-arid and cold, with marked seasonality: a dry season from May to November and a rainy season from December to April. In the study area, guinea pigs are reared under a family-commercial production system with a basic level of technification. Animals are kept in pens and fed twice daily with a mixed diet based on green forage, mainly alfalfa (*Medicago sativa*), ryegrass (*Lolium perenne*), and oats forage (*Avena sativa*). This diet is supplemented with ground plant-based inputs in intermediate particle sizes, comparable to balanced concentrates used in animal nutrition, including alfalfa, soybean, maize, wheat, and cottonseed meal, in addition to vitamin and mineral supplements. From a sanitary perspective, limitations are evident due to inadequate management practices and the lack of technical knowledge, particularly regarding pen cleaning, which is performed approximately every three months due to fecal accumulation. Moreover, in forage cultivation areas used for feeding guinea pigs, some farmers allow other domestic species, such as alpaca, cattle and sheep, to graze.

### 2.2 Sample collection

**Fig. 1:**
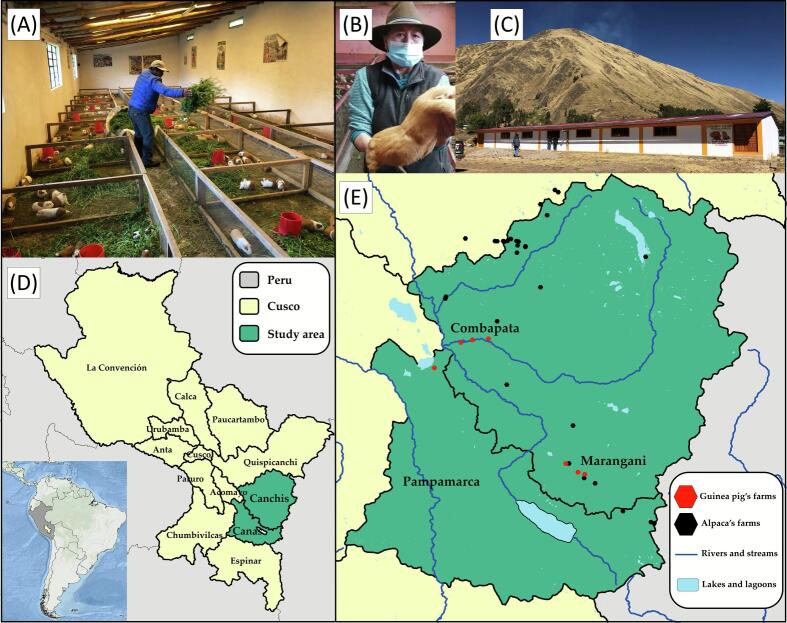
Study area and guinea pig sampling sites in Cusco, Peru. (A) Guinea pig small-scale family farming system. (B) Farmer holding a guinea pig. (C) Exterior of a representative farm. (D) The location of Canchis and Canas provinces in the Cusco region, Peru. (E) Distribution of guinea pig's farms (n = 7), alpacas and water body sources in Marangani, Combapata, and Pampamarca districts in Cusco region, Perú.

Based on the leading status of guinea pig production two provinces were selected for the investigation: Canchis was represented by six farms and Canas by a single farm (Fig. 1). Farms and animals included in this study were selected through convenience sampling based on logistical accessibility and the willingness of smallholder producers to participate. The seven farms represented traditional small-scale production systems (<100 animals), characterized by rustic housing, locally sourced forage, and minimal sanitary management. A total of 34 intestinal tissue samples were collected from adult guinea pigs (>6 months old) showing apparent gastrointestinal lesions at necropsy during the raining season.

### 2.3 RNA extraction

RNA was extracted from 10% tissue suspensions following the method of Rojas et al. (2019) [12], with minor modifications. Briefly, sections of the duodenum, jejunum, and ileum (approximately 0.5 cm), including their contents, were homogenized and transferred into 10 ml Falcon tubes. Each suspension was adjusted to a final volume of 5 ml with 20% phosphate-buffered saline (PBS, pH 7.2) and centrifuged at 3000 ×*g* for 10 min. RNA was then extracted from the supernatants using the Nucleic Acid Extraction Reagent Kit (Lifotronic Technology Co., Ltd., Shenzhen, China) in combination with the Auto-Pure32A automated extractor (Nucleic Acid Purification System, ALLSHENG, Zhejiang, China), according to the manufacturer's instructions. Samples were stored at −80 °C until processing.

### 2.4 Reverse transcription polymerase chain reaction (RT-PCR) and nested PCR

RNA extracts were screened for Coronavirus (CoV), Rotavirus A (RVA), *Mammalian Orthoreovirus* (MRV), and Kobuvirus (KoV). Complementary DNA (cDNA) synthesis was performed using antisense primers specific to each virus (Table 1) and the GoScript™ Reverse Transcription System (Promega, Madison, WI, USA). Before reverse transcription, samples were subjected to an initial denaturation step at 95 °C for 5 min.

**Table 1:** Primers used in the RT-PCR and nested PCR assays for Coronavirus (CoV), Rotavirus A (RVA), *Mammalian Orthoreovirus* (MRV), and Kobuvirus (KoV) detection.

Virus	Target gene	Assay	Primer	Primer sequence 5′ – > 3’	Amplicon size (bp)	Reference
CoV	RdRp^a^	RT-PCR	Cor-FW	ACWCARHTVAAYYTNAARTAYG	251	[13]
			Cor-RV	TCRCAYTTDGGRTARTCCCA		
β-CoV	RdRp	Nested PCR	Beta.CoV·F	ATTAGTGCWAAGAATAGAGCYCGCA	227	[14]
			Beta.Cov.R	TCACAYTTWGGRTARTCCCADCCCA		
Embecovirus	RdRp	Nested PCR	CV2U·F	TACTATGACTGGCAGAATGTTTC	136	[15]
			CV2L.R	AACATCTTTAATAAGGCGRCGTA		
RVA	NSP5^b^	RT-PCR	GEN_NSP5.1F	GGTTTTAAAGCGCTACAG	667	[16]
			GEN_NSP5.2R	GGTCACAAAACGGGAGT		
RVA	NSP5	Nested PCR	MAX_NSP5.1FM	CGTCAACTCTTTCTGGAAAATCTA	562	[17]
			MAX_NSP5.4RM	GTGGGGAGCTCCCTAGT		
MRV	RdRp^a^	RT-PCR	MRV-FM	CCNATATCNGGAATGCAGAA	181	[14]
			MRV-RM	TCCATCATCGTRCTATTRTTNGC		
KoV	RdRp^a^	RT-PCR	UNIV-kobu-F	TGGAYTACAARTGTTTTGATGC	216	[18]
			UNIV-kobu-R	ATGTTGTTRATGATGGTGTTGA		

RdRp^a^ = RNA-dependent RNA polymerase, NSP5^b^ = Non-structural protein 5, bp: base pair.

Molecular protocols were developed based on previously described methods: Moes et al. [13] for CoV, Castilla et al. [14] for β-CoV, and Bezerra et al. [15] for *Embecovirus*. Protocols of Matthijnssens et al. [16] for PCR and Rojas et al. [17] for nested PCR were applied for RVA detection. For MRV, we followed the protocol of Castilla et al. [14]. Finally, KoV detection was performed according to the method described by Reuter et al. [18]. The PCR and nested PCR were performed using the GoTaq® Green Master Mix (Promega, Madison, WI, USA) in a T100 thermal cycler (Bio-Rad Laboratories, Hercules, CA, USA). Amplification products were separated on 1.5% (*w*/*v*) agarose gel electrophoresis, stained with ethidium bromide, and visualized under UV light. A 100 bp DNA ladder (Promega, Madison, WI, USA) was used as a molecular-weight size marker.

### 2.5 Positives controls

Each test was validated using two controls. The positive control consisted of viral strains previously isolated in cell culture and confirmed by sequencing (GenBank accession numbers KX266949 and KX266944 for CoV, MN200216 for MRV, and KT935485 for RVA); for KoV, a synthetic control was obtained from Integrated DNA Technologies (IDTdna, Coralville, USA). The negative control was a no-template control (NTC), consisting of sterile water and master mix to monitor potential contamination.

### 2.6 Statistical analysis

The data were processed and analyzed using STATA version 16.1. Frequencies and their corresponding 95% confidence intervals were calculated using the binomial exact method (default setting in the software).

### 2.7 Nucleotide sequencing and analysis

A total of 21 PCR amplicons (CoV, *n* = 5; RVA, *n* = 10; MRV, *n* = 3; KoV, n = 3) were chosen for Sanger sequencing based on their PCR results (samples with the highest number of different viruses detected and band intensity). Sequencing was performed by Macrogen Inc. (Seoul, Republic of Korea), and raw data were processed using EditSeq, MegAlign, and SeqMan with DNAstar Lasergene software package (Version 7.0, DNASTAR, Madison, WI, USA). Phylogenetic analysis was performed in the MEGA12 software, and dendrograms were constructed using the maximum likelihood method based on the Kimura 2-parameter model [19] with 1000 bootstrap replicates. Sequences were compared with nucleotide reference sequences obtained from GenBank to confirm the PCR assays. In the case of RVA, the genotype H classification was based on Matthijnssens et al. [16] using 91% nucleotide identity as the cut-off value. The sequences were deposited into NCBI GenBank under accession numbers: CoV (PX458881-PX458885), KoV (PX458886-PX458888), MRV (PX458889-PX458891), RVA (PX458892-PX458901).

## 3 Results

### 3.1 Molecular detection of zoonotic RNA viruses

**Fig. 2:**
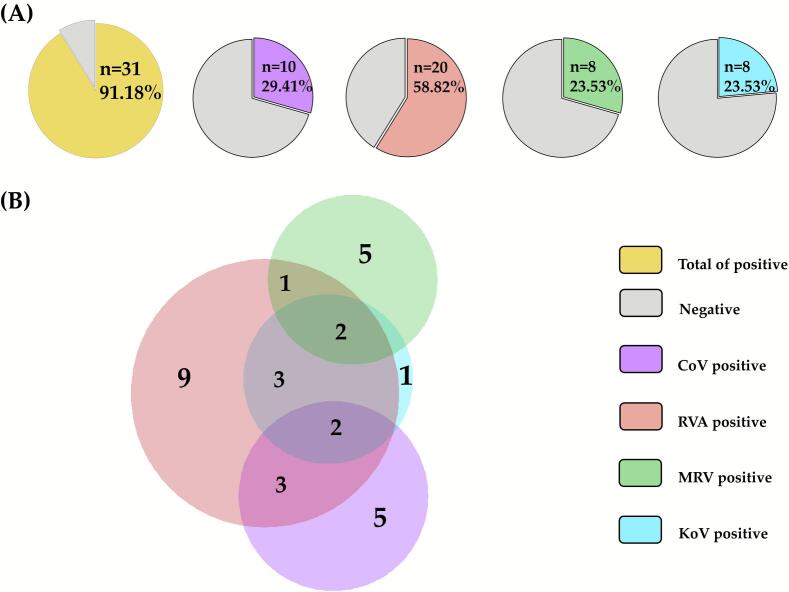
(A) Proportion of positive samples and (B) number of single, double, and triple infections for each of the different investigated enteric RNA viruses (Coronavirus - CoV, Rotavirus A - RVA, *Mammalian Orthoreovirus* - MRV, and Kobuvirus - KoV) in 34 guinea pigs from Cusco region, Peru.

Zoonotic RNA virus detection and types of infections in guinea pigs (*n* = 34) are shown in Fig. 2. Overall, 91.18% (31/34, 95% CI: 76.32–98.14%) of samples tested positive for at least one virus: Rotavirus A (RVA) was the most frequent detected in 58.82% (20/34, 95% CI: 40.70–75.35%) of samples, followed by Coronavirus (CoV) in 29.41% (10/34, 95% CI: 15.10–47.48%), and both *Mammalian orthoreovirus* (MRV) and Kobuvirus (KoV) in 23.53% (8/34, 95% CI: 10.75–41.47%), each (Fig. 2A). Regarding types of infection, single-virus detections accounted for 20 cases (64.52%), dominated by RVA (*n* = 9). Co-infections were identified in 11 cases (35.48%): double infections (*n* = 7), most commonly RVA-CoV and RVA-KoV (three cases each), and triple infections (*n* = 4), which involved RVA with KoV either CoV or MRV (Fig. 2B). KoV detection was predominantly associated with co-infections (7/8) rather than single infections.

### 3.2 Phylogenetic analysis

**Fig. 3:**
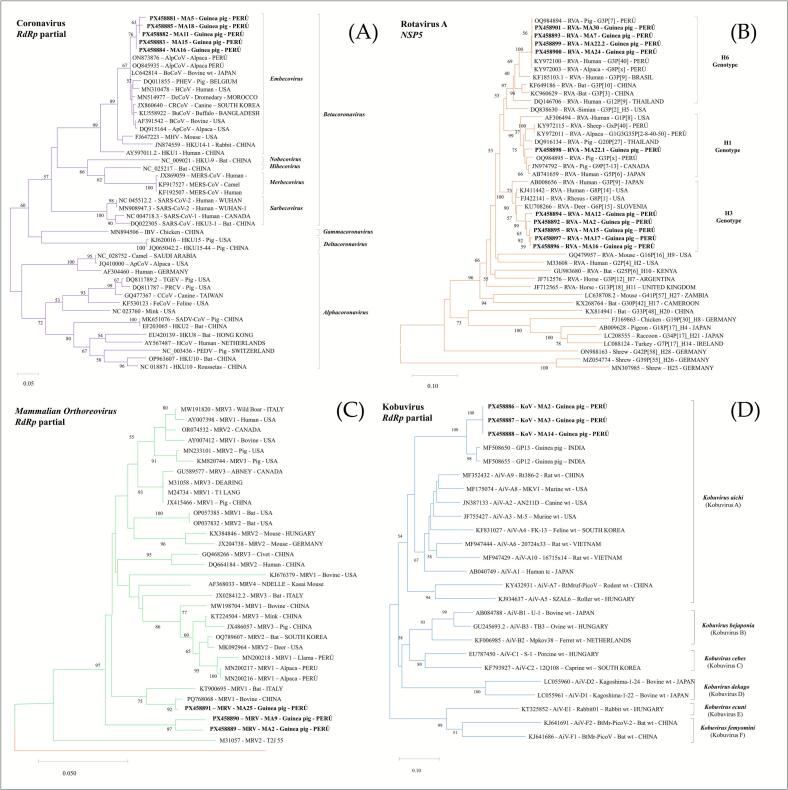
Phylogenetic trees were constructed using the maximum likelihood method based on partial nucleotide sequences of enteric RNA viruses: (A) Coronavirus, CoV in purple; (B) Rotavirus A, RVA in orange; (C) *Mammalian orthoreovirus*, MRV in green; and (D) Kobuvirus, KoV in blue. Distances were corrected with the Kimura 2-parameter model with 1000 bootstrap replicates. Bootstrap values >50% are given at branch nodes. The distance scale reflects substitutions per site. Strains generated in this study are indicated in black. (For interpretation of the references to colour in this figure legend, the reader is referred to the web version of this article.)

Phylogenetic analysis of the partial sequences of the *RdRp* gene (136 bp) of CoV revealed that our strains shared 97.8–100% nucleotide identity each other and clustered within *Embecovirus* subgenus (85.3 to 100% nucleotide identity) of the *Betacoronavirus* genus (Fig. 3A). The CoV sequences showed higher identity (>96.3%) with sequences reported in alpaca (ON873876-Peru and OQ845935-Peru) and cattle (LC642814-Japon).

The complete *NSP5* gen (562 bp) of RVA sequences of the study showed identity from 83% to 99.8% at the nucleotide level (Fig. 3B). The phylogenetic tree illustrated that samples clustered into three distinct clades within the H genotype. Four sequences clustered within the H6 genotype (>91.6% nucleotide identity) with previously reported Peruvian strains. Five sequences belong to the H3 genotype (97.3 to 99.7% nucleotide identity). One of the study sequences was grouped differently from the remaining sequences within the H1 genotype (91.5 to 99.8% nucleotide identity). Evidence of co-infection with genotypes H1 and H6 was detected in one sample.

The partial *RdRp* gene sequences obtained in this study (181 bp for MRV and 216 bp for KoV) were analyzed and compared with reference sequences. The MRV sequences showed nucleotide identity from 89.6 to 96.7% among themselves. When compared with reference strains representing serotypes 1, 2, 3, and 4, identities varied between 78.7 and 99.5% (Fig. 3C). KoV sequences revealed that all sequences were 100% identical to each other and shared 93.1% nucleotide identity with previously reported guinea pig strains from India (MF508650 and MF508655). These sequences formed a monophyletic clade within *Kobuvirus aichi* (Kobuvirus A), with 64.5 to 77.9% nucleotide identity (Fig. 3D).

## 4 Discussion

Although guinea pigs (*Cavia porcellus*) are widely used as experimental models in biomedical research, kept as pets in many Western countries and represent an important source of animal protein in the Andean region, most virological investigations have been limited to controlled laboratory conditions, and field-based investigations in naturally raised animals remain scarce. Therefore, this study provides the first molecular evidence of multiple zoonotic RNA viruses circulating in guinea pigs raised under small-scale family farming systems in Peru, offering novel insights into viral circulation within traditional farming in Andean environments. The high overall frequency (91.18%) observed in this study highlights a substantial viral burden among animals living in close proximity to humans. Comparable studies in other domestic species, such as pigs and cattle, have reported similar frequencies of RNA viruses, typically between 76% and 88%, depending on environmental, sanitary, and management factors [20], [21]. The elevated frequency detected here may be related to the traditional husbandry practices in the Andean region, which are characterized by poor housing conditions, frequent interspecies contact, and limited biosecurity. Such circumstances can facilitate viral maintenance and transmission and may promote the occurrence of co-infections among animals sharing feeding areas or confined spaces [12].

In addition, all samples analyzed were obtained from animals presenting intestinal lesions at necropsy, which likely contributed to the high detection frequency of enteric RNA detected. This conditional sampling of diseased tissues may have introduced a detection bias and therefore should be considered when interpreting the reported frequencies, as they may not reflect virus prevalence in clinically healthy animals. Comparable studies in the Andean region have focused on alpacas and llamas, as no virological investigations of guinea pigs have been reported at the regional, national or continental level. High detection frequencies of enteric RNA viruses have also been documented in animals with gastrointestinal disease from the Andean region. For example, RVA in alpacas 28.6%, 47.5% in llamas and 100% in sheep [12]. Similarly, a study of 50 preweaning with dehydrating diarrhea from an Andean herd in Cusco reported RVA and CoV frequencies of 32% and 40%, respectively [22]. Recently, it was reported that 95% of fecal samples from alpacas and llamas were positive for at least one RNA virus (CoV, MRV, RVA) with all animals showing clinical signs of diarrhea [14]. These findings support the high virus frequency observed in our study with animals exhibiting gastrointestinal pathology in the Andean region.

RVA was the most frequently detected virus, followed by CoV, MRV, and KoV in the analyzed guinea pig samples. Co-infections were detected in nearly one-third of the positive samples (11/31), suggesting that guinea pigs may serve as important reservoirs for the persistence and transmission of diverse zoonotic viruses within small-scale family farming systems. Similar co-infection patterns have been reported in other domestic species, such as pigs and cattle, where the frequency of mixed infections ranges from 37% to 40% in Egypt, in semi-extensive and indoor closed farms [20], [21]. Co-infection is a major driver of viral evolution, as homologous and heterologous recombination events may occur during RNA replication. Such genetic exchanges may result in the emergence of novel viral lineages with modified pathogenicity or even host specificity. One example is the novel coronavirus Ro-BatCoVGCCDC1, discovered in bats from China, which likely emerged through recombination between an ancestral *Coronavirus* and an *Orthoreovirus* [23]. Another well-documented case of inter-family recombination involves the hemagglutinin-esterase (HE) gene of *Betacoronavirus* group A, which appears to have been derived from an ancestral influenza C virus [24]. These examples illustrate the remarkable evolutionary plasticity of RNA viruses and emphasize the importance of studying animal species in rural areas where co-infections are common.

Overall, the phylogenetic analysis of enteric RNA viruses detected in guinea pigs provides strong evidence of a diverse and complex enteric virome circulating in this environment. Most sequences clustered with viral strains reported from other continents, with only limited representations of local or Andean references. This pattern is largely explained by the limited availability of locally generated viral sequences from Andean region in public databases. In particular, publicly available genomic data from South America remain scarce, especially from high-altitude Andean ecosystems, therefore to address this limitation all available regional reference sequences were incorporated into the phylogenetic analysis. Notably, molecular studies conducted in this region have focused mainly on South American Camelids, such as alpacas and llamas [12], [14], [22].

The close relationship of RVA and CoV with previously reported strains in alpacas of the same region suggests possible interspecies transmission [12], [25]. Guinea pig farms are frequently located in close proximity to alpaca holdings, and shared water sources-such as rivers, streams, and irrigation channels-are routinely used for crop cultivation (Fig. 1). The potential transmission between alpacas and guinea pigs in this region may be explained by indirect contact through environmental contamination, particularly using feces from one species as organic fertilizer for forage consumed by the other. This agricultural practice could facilitate viral persistence in the environment and promote interspecies transmission through the fecal–oral route. In the case of RVA, the detection of H1, H3 and H6 genotypes within the sampled population; suggests the persistence and continued circulation of local RVA variants, reflecting genetic heterogeneity. Detecting mixed infection involving genotypes H1 and H6 in a single sample provides molecular evidence of co-infection and supports the possibility of reassortment events in guinea pigs. In developing countries, limited access to safe drinking water, restricted limited health services and close cohabitation with farm animals have been associated with an increased risk of zoonotic rotavirus transmission [26]. Consequently, such reassortments are commonly detected in developing regions of Africa, Asia, and Latin America [27].

Molecular analysis confirmed the presence of *Mammalian orthoreovirus* (MRV) and *Kobuvirus aichi* (formerly Aichivirus A) in the guinea pig population. MRV is a neglected zoonotic virus commonly detected in early childhood, where infections are often asymptomatic. When clinical signs occur, MRV infection in humans manifests mainly as coryza, pharyngitis, cough, or gastroenteritis [28]. Certain strains, such as MRV2Tou05 detected in children with encephalopathy in France, showed close genetic similarity to porcine and human strains [29]. These findings emphasize the need to identify the MRV serotypes circulating in guinea pigs to better understand their potential role in zoonotic transmission.

Regarding KoV, the human Aichi virus (AiV-1) is transmitted by the fecal-oral route, mainly through contaminated food or water, and is considered an emerging pathogen associated with environmental contamination. The risk of infection is higher in developing countries due to inadequate sanitation and poor-quality drinking water, particularly in rural areas [30], [31]. In our study, KoV was detected more frequently in co-infections than in single infections, suggesting possible viral interactions that warrant further investigation. Therefore, continued molecular characterization of KoV is therefore essential to determine its specific type and genotype, as our phylogenetic analysis revealed a monophyletic clade closely related to Indian strains but distinct from the ten currently recognized *Kobuvirus aichi* types (AiV-1 to AiV-10).

Our findings revealed the coexistence of multiple zoonotic RNA viruses within guinea pigs raised under traditional small-scale farming systems in the Peruvian Andes. The high virus detection frequency together with the presence of co-infections and close phylogenetic relationships with strains previously reported in livestock and humans situates guinea pigs within a broader One Health framework, where interconnected human-animal-environment interfaces drive viral circulation. In smallholder farming settings, frequent human contact, multispecies cohabitation, and limited biosecurity create ecological conditions that favor viral maintenance and interspecies spillover. Consequently, continuous molecular surveillance and genomic characterization of these viruses are essential to understand their evolutionary dynamics, transmission pathways, and potential public health risks in rural Andean ecosystems. Future studies should also include environmental sampling to assess potential contamination and human exposure during guinea pig management. Finally, expanded local sequencing efforts, combined with phylogeographic and molecular clock analyses, will be critical to strengthen One Health-oriented surveillance strategies in Andean rural farming systems.

## 5 Conclusion

This study provides compelling evidence that guinea pigs raised under traditional small-scale farming systems in the Peruvian Andes harbor diverse zoonotic RNA viruses. The observed viral diversity and phylogenetic links with human and livestock strains suggest that these animals may play an underestimated role in maintaining and transmitting zoonotic viruses in rural ecosystems.

## CRediT authorship contribution statement

**Abel E. Quispe:** Writing – review & editing, Writing – original draft, Methodology, Investigation, Conceptualization. **Renzo Vera:** Methodology, Conceptualization. **Josimar Quiñones:** Methodology, Conceptualization. **José Angulo-Tisoc:** Writing – review & editing, Methodology. **César Lázaro:** Writing – review & editing, Methodology. **Alberto Manchego:** Methodology. **Milagros Lostaunau:** Methodology. **Edgar Valdez:** Methodology. **Miguel Rojas:** Writing – review & editing, Validation, Software, Resources, Investigation, Funding acquisition. **Dennis A. Navarro-Mamani:** Writing – review & editing, Writing – original draft, Validation, Supervision, Methodology, Conceptualization.

## Ethics statement

The animal research ethics committee CEBA N° 2024–22 of the Facultad de Medicina Veterinaria, Universidad Nacional Mayor de San Marcos (FMV-UNMSM), Lima, Peru, approved the present study.

## Funding

This work was financially supported by the Programa Nacional de Investigación y Estudios Avanzados (PROCIENCIA) – CONCYTEC en el marco de la convocatoria de “Proyecto de Investigación Básica 2024-01” (grant number PE501088213-2024).

## Declaration of competing interest

The authors declare that they have no competing financial interests or personal relationships that could have influenced the work reported in this paper.

## Data Availability

Data will be made available on request.

## References

[1] Johnson C.K., Hitchens P.L., Pandit P.S., Rushmore J., Evans T.S., Young C.C.W., Doyle M.M. (2020). Global shifts in mammalian population trends reveal key predictors of virus spillover risk. Proc. R. Society Biol. Sci..

[2] Gupta S., Kaur R., Sohal J.S., Singh S.V., Das K., Sharma M.K., Singh J., Sharma S., Dhama K. (2024). Countering zoonotic diseases: current scenario and advances in diagnostics, monitoring, prophylaxis and therapeutic strategies. Arch. Med. Res..

[3] Magouras I., Brookes V.J., Jori F., Martin A., Pfeiffer D.U., Dürr S. (2020). Emerging zoonotic diseases: should we rethink the animal–human interface?. Front. Vet. Sci..

[4] Welburn S.C., Beange I., Ducrotoy M.J., Okello A.L. (2015). The neglected zoonoses-the case for integrated control and advocacy. Clin. Microbiol. Infect..

[5] Lu M., He W.T., Pettersson J.H.O., Baele G., Shi M., Holmes E.C., He N., Su S. (2023). Zoonotic risk assessment among farmed mammals. Cell.

[6] Tomley F.M., Shirley M.W. (2009). Livestock infectious diseases and zoonoses. Philos. Trans. R. Soc. B.

[7] Pulliam J.R.C., Dushoff J. (2009). Ability to replicate in the cytoplasm predicts zoonotic transmission of livestock viruses. J. Infect. Dis..

[8] Zhao J., Wan W., Yu K., Lemey P., Pettersson J.H.O., Bi Y., Lu M., Li X., Chen Z., Zheng M., Yan G., Dai J.J., Li Y., Haerheng A., He N., Tu C., Suchard M.A., Holmes E.C., He W.T., Su S. (2024). Farmed fur animals harbour viruses with zoonotic spillover potential. Nature.

[9] Rodriguez-Pazmiño A.S., Zambrano-Mila M., Salas-Rueda M., Cáceres-Orellana M.V., Buele-Chica D., Barrera-Barroso L., Rivera-Olivero I., Cardenas W.B., Orlando S.A., Parra-Vera H., Garcia-Bereguiain M.A. (2025). Respiratory pathogens carriage in guinea pigs raised as livestock in Ecuador: a proxy to study a neglected reservoir for zoonotic transmission in the Andean region. Acta Trop..

[10] Donoso G., Galecio J.S., Fuentes-Quisaguano O.G., Pairis-Garcia M. (2025). Guinea pig meat production in South America: reviewing existing practices, welfare challenges, and opportunities. Anim. Welf..

[11] Ortiz-Oblitas P., Florián-Alcántara A., Estela-Manrique J., Rivera-Jacinto M., Hobán-Vergara C., Murga-Moreno C. (2021). Characterization of the breeding of guinea pigs in three provinces of the Cajamarca region, Peru. Rev. Invest. Vet. Peru.

[12] Rojas M., Dias H., Gooncalves J.L., Manchego A., Rosadio R., Pezo D., Santos N. (2019). Genetic diversity and zoonotic potential of rotavirus a strains in the southern Andean highlands, Peru. Transbound. Emerg. Dis..

[13] Moës E., Vijgen L., Keyaerts E., Zlateva K., Li S., Maes P., Pyrc K., Berkhout B., van der Hoek L., Van Ranst M. (2005). A novel pancoronavirus RT-PCR assay: frequent detection of human coronavirus NL63 in children hospitalized with respiratory tract infections in Belgium. BMC Infect. Dis..

[14] Castilla D., Escobar V., Ynga S., Llanco L., Manchego A., Lázaro C., Navarro D., Santos N., Rojas M. (2021). Enteric viral infections among domesticated south American camelids: first detection of mammalian orthoreovirus in camelids. Animals.

[15] Bezerra J., Brandão P., Petinatti S., Varaschin M., Wouters F., Villarreal L., Jerez J., Costa G. (2009). Outbreak of diarrhea in cows from a dairy herd in the southern region of Minas Gerais state: detection of bovine coronavirus in the feces. Arquivo Brasileiro Medicina Veterinária Zootecnia.

[16] Matthijnssens J., Ciarlet M., Heiman E., Arijs I., Delbeke T., McDonald S.M., Palombo E.A., Iturriza-Gómara M., Maes P., Patton J.T., Rahman M., Van Ranst M. (2008). Full genome-based classification of rotaviruses reveals a common origin between human Wa-like and porcine rotavirus strains and human DS-1-like and bovine rotavirus strains. J. Virol..

[17] Rojas M.A., Gonçalves J.L.S., Dias H.G., Manchego A., Santos N. (2017). Identification of two novel rotavirus a genotypes, G35 and P[50], from Peruvian alpaca faeces. Infect. Genet. Evol..

[18] Reuter G., Boldizsár Á., Pankovics P. (2009). Complete nucleotide and amino acid sequences and genetic organization of porcine kobuvirus, a member of a new species in the genus Kobuvirus, family Picornaviridae. Arch. Virol..

[19] Kimura M. (1980). A simple method for estimating evolutionary rates of base substitutions through comparative studies of nucleotide sequences. J. Mol. Evol..

[20] Capai L., Piorkowski G., Maestrini O., Casabianca F., Masse S., de Lamballerie X., Charrel R.N., Falchi A. (2022). Detection of porcine enteric viruses (Kobuvirus, Mamastrovirus and Sapelovirus) in domestic pigs in Corsica, France. PLoS One.

[21] Mohamed F., Mansour S., El-Araby I., Mor S., Goyal S. (2017). Molecular detection of enteric viruses from diarrheic calves in Egypt. Arch. Virol..

[22] Rojas M., Manchego A., Rocha C.B., Fornells L.A., Silva R.C., Mendes G.S., Dias H.G., Sandoval N., Pezo D., Santos N. (2016). Outbreak of diarrhea among preweaning alpacas (Vicugna pacos) in the southern Peruvian highland. J. Infect. Dev. Ctries..

[23] Huang C., Liu W., Xu W., Jin T., Zhao Y., Song J., Shi Y., Ji W., Jia H., Zhou Y., Wen H., Zhao H., Liu H., Li H., Wang Q., Wu Y., Wang L., Liu D., Liu G., Yu H., Holmes E., Lu L., Gao G. (2016). A bat_derived putative cross family recombinant coronavirus with a reovirus gene. PLoS Pathog..

[24] De Groot R.J. (2006). Structure, function and evolution of the hemagglutinin-esterase proteins of corona- and toroviruses. Glycoconj. J..

[25] Llanco L., Retamozo K., Oviedo N., Manchego A., Lázaro C., Navarro-Mamani D.A., Santos N., Rojas M. (2023). Co-circulation of multiple coronavirus genera and subgenera during an epizootic of lethal respiratory disease in newborn alpacas (*Vicugna pacos*) in Peru: first report of bat-like coronaviruses in alpacas. Animals.

[26] Cook N., Bridger J., Kendall K., Iturriza M., El-Attar L., Gray J. (2004). The zoonotic potential of rotavirus. J. Inf. Secur..

[27] Bányai K., László B., Duque J., Steele D., Nelson A., Gentsch J., Parashar U. (2012). Systematic review of regional and temporal trends in global rotavirus strain diversity in the pre rotavirus vaccine era. Vaccine.

[28] Yamamoto S.P., Motooka D., Egawa K., Kaida A., Hirai Y., Kubo H., Motomura K., Nakamura S., Iritani N. (2020). Novel human reovirus isolated from children and its long-term circulation with reassortments. Sci. Rep..

[29] Ouattara L.A., Barin F., Barthez M.A., Bonnaud B., Roingeard P., Goudeau A., Castelnau P., Vernet G., Paranhos-Baccalà G., Komurian-Pradel F. (2011). Novel human reovirus isolated from children with acute necrotizing encephalopathy. Emerg. Infect. Dis..

[30] Abdelqader R., Hasan H., Shuqair D., Zueter A., Albakri K., Ghanem M. (2025). Global epidemiology, genotype distribution and coinfection rate of human Aichi virus: a systematic review. J. Inf. Chemother..

[31] Sdiri-Loulizi K., Khachou A., Lemaire S., Bour J., Ayouni S., Kaplon J., Sakly N., Aouni M., Belliot G., de Rougemont A. (2025). Persistence of human Aichi virus infectivity from raw surface water to drinking water. Appl. Environ. Microbiol..

